# Abbreviated perioperative fasting management for elective fresh fracture surgery: guideline adherence analysis

**DOI:** 10.1186/s12891-022-05574-5

**Published:** 2022-07-20

**Authors:** Zhi-jian Sun, Xu Sun, Yan Huo, Meng Mi, Gui-ling Peng, Chun-ling Zhang, Yao Jiang, Yan Zhou, Xia Zhao, Ting Li, Xin-bao Wu

**Affiliations:** 1 Department of Orthopedic Trauma, Beijing Jishuitan Hospital, Beijing, 100035 China; 2 Department of Anesthesiology, Beijing Jishuitan Hospital, Beijing, 100035 China; 3 Department of Nutriology, Beijing Jishuitan Hospital, Beijing, 100035 China

**Keywords:** Perioperative fasting, Compliance, Fresh fractures, Enhanced recovery after surgery

## Abstract

**Background:**

Long-term fasting for elective surgery has been proven unnecessary based on established guidelines. Instead, preoperative carbohydrate loading 2 h before surgery and recommencing oral nutrition intake as soon as possible after surgery is recommended. This study was performed to analyze the compliance with and effect of abbreviated perioperative fasting management in patients undergoing surgical repair of fresh fractures based on current guidelines.

**Methods:**

Patients with fresh fractures were retrospectively analyzed from the prospectively collected database about perioperative managements based on enhanced recovery of surgery (ERAS) from May 2019 to July 2019 at our hospital. A carbohydrate-enriched beverage was recommended up to 2 h before surgery for all surgical patients except those with contraindications. Postoperatively, oral clear liquids were allowed once the patients had regained full consciousness, and solid food was allowed 1 to 2 h later according to the patients’ willingness. The perioperative fasting time was recorded and the patients’ subjective comfort with respect to thirst and hunger was assessed using an interview-assisted questionnaire.

**Results:**

In total, 306 patients were enrolled in this study. The compliance rate of preoperative carbohydrate loading was 71.6%, and 93.5% of patients began ingestion of oral liquids within 2 h after surgery. The median (interquartile range) preoperative fasting time for liquids and solids was 8 (5.2–12.9) and 19 (15.7–22) hours, respectively. The median postoperative fasting time for liquids and solids was 1 (0.5–1.9) and 2.8 (2.2–3.5) hours, respectively. A total of 70.3% and 74.2% of patients reported no thirst and hunger during the perioperative period, respectively. Logistic regression analysis showed that the preoperative fasting time for liquids was an independent risk factor for perioperative hunger. No risk factor was identified for perioperative thirst. No adverse events such as aspiration pneumonia or gastroesophageal reflux were observed.

**Conclusions:**

In this study of a real clinical practice setting, abbreviated perioperative fasting management was carried out with high compliance in patients with fresh fractures. The preoperative fasting time should be further shortened to further improve patients’ subjective comfort.

## Background

Perioperative fasting is required for elective surgery to prevent aspiration pneumonia, nausea, vomiting, and dysphagia [1]. However, long-term fasting not only worsens patients’ discomfort by increasing thirst, hunger, anxiety, and fatigue but also deteriorates insulin resistance [2–4]. Many guidelines have recommended access to clear liquids and solids for up to 2 and 6 h, respectively, before elective surgery [5–8]. Furthermore, a preoperative carbohydrate-enriched beverage has been highly recommended to reduce patients’ discomfort, the impact of postoperative insulin resistance, and the length of hospital stay [8–11].

The consensus on postoperative fasting is that interruption of oral nutrition intake after surgery is usually unnecessary. Oral liquids and food should be recommenced as soon as possible according to the patients’ tolerance [7, 8, 12]. However, because of significant variations among types of surgeries performed, the exact time at which oral intake should be recommenced postoperatively is absent in common guidelines. In 2019, the Chinese guideline of perioperative fasting management in orthopedic surgery recommended resuming oral clear liquids once patients have regained their awareness (as assessed by the Steward score) and then transiting to a normal diet in the following 1 to 2 h, considering that the gastrointestinal tract is seldom involved in orthopedic surgeries [13].

Nevertheless, compliance with abbreviated perioperative fasting is usually poor because of uncertainty regarding the operation starting time, surgeons’ and anesthetists’ viewpoints, patients’ willingness, and similar influencing factors [14–17]. Moreover, most studies have only reported the preoperative fasting status; postoperative oral nutrition recommencement has rarely been studied [18–20]. Thus, we conducted this study to analyze the compliance with and effect of abbreviated perioperative fasting management in patients undergoing surgical treatment of fresh fractures.

## Methods

### Participants

Patients with fresh fractures were retrospectively analyzed from the prospectively collected database about perioperative managements based on enhanced recovery of surgery (ERAS) from May 2019 to July 2019 at our hospital according to the following criteria.

The inclusion criteria were an age of ≥ 14 years with no sex limitation; an upper or lower extremity, pelvic, or acetabular fracture with a single part; a fresh fracture (< 3 weeks); and voluntary participation in this study with provision of written informed consent.

The exclusion criteria were multiple trauma; open fractures; mental disorders, alcohol dependence, or a history of drug abuse; pregnancy or lactation; allergic constitution or allergy to a variety of drugs; and lack of suitability for this study as judged by researchers.

This study was approved by the ethics committee of Beijing Jishuitan Hospital (201,807–12). And the research was performed in accordance with relevant guidelines. Informed consent was obtained from all participants.

### Perioperative fasting protocol

Abbreviated perioperative fasting management was conducted according to the concept of enhanced recovery after surgery (ERAS), which was introduced at our trauma center in 2016. Clear liquids were recommended up to 2 h before surgery for all surgical patients except those with delayed gastric emptying associated with conditions such as emergency surgery, gastrointestinal obstruction disease, upper gastrointestinal cancer, morbid obesity, pregnancy, gastroesophageal reflux, diabetes mellitus, and inability to engage in oral feeding. A carbohydrate-enriched beverage containing 12.5% maltodextrin (made by the nutritional department of our hospital) was highly recommended [5, 8]. Patients were allowed to drink 800 mL of this beverage the night before surgery and no more than 400 mL the day of surgery up to 2 h prior to surgery. Patient receipt of the carbohydrate-enriched beverage preoperatively on the day of surgery was defined as compliance with preoperative carbohydrate loading. And patients without contraindications did not receive preoperative carbohydrate loading was defined as true non-compliance. Postoperatively, the anesthetists assessed the patients’ consciousness using the Steward score, and oral clear liquids were allowed when the Steward score reached ≥ 5. Solid food was then allowed 1 to 2 h later according to the patients’ willingness [4]. Patients regained oral liquids or solids within 2 h after surgery were defined as compliance with abbreviated postoperative fasting.

### Data collection

All data were collected prospectively. The collected data included patients’ basic information [age, sex, body mass index (BMI), fracture type (upper extremity fracture, lower extremity fracture, or pelvic/acetabular fracture), comorbidities (hypertension, diabetes mellites, or other conditions)] and surgical information [anesthesia type (general anesthesia and spinal/regional blocking anesthesia), surgery type (open reduction with internal fixation, closed reduction with internal fixation, and others), and surgical time]. The perioperative fasting times, including the preoperative liquid fasting time, preoperative solid fasting time, postoperative liquid fasting time, and postoperative solid fasting time, were also collected. The total fasting time for liquids and solids was then calculated. Patients’ subjective comfort with respect to thirst, hunger, nausea and vomiting (ranked as none, some, or severe) was assessed using an interview-assisted questionnaire. Adverse events, such as aspiration pneumonia or gastroesophageal reflux, were recorded. The diagnosis of such events was made by chest radiographs/CT scan or esophagoscope for clinical suspicious patients.

### Statistical analysis

Statistical analysis was performed using IBM SPSS Statistics for Windows, Version 25.0 (IBM Corp., Armonk, NY, USA). Quantitative data are presented as mean ± standard deviation or median (interquartile range), and categorical data are presented as number (percentage). For group comparisons of quantitative data, Student’s t test and the Mann–Whitney U test were used for normally and non-normally distributed data, respectively. For group comparisons of categorical data, the chi-square test was used. Taking patients’ subjective comfort with respect to thirst and hunger as a dependent variable, logistic regression tests were performed to identify relevant risk factors. A *P* value of < 0.05 was considered statistically significant.

## Results

### Compliance analysis

In total, 306 patients were enrolled in this study, and the basic information was illustrated in Table 1. The patients comprised 183 (59.8%) men and 123 (40.2%) women with a mean age of 43.7 ± 16.0 years. 42 (13.7%) patients received general anesthesia, and spinal/reginal blocking was performed for the other 264 patients (86.3%). A total of 219 patients underwent preoperative carbohydrate loading the day before and the day of surgery (compliance rate of 71.6%). Ten (3.3%) patients did not drink the carbohydrate-enriched beverage because of contraindications. Seventy-seven (25.2%) patients had true non-compliance with abbreviated preoperative fasting. For postoperative fasting, 286 (93.5%) patients regained oral fluids within 2 h after surgery. No adverse events, such as aspiration pneumonia or gastroesophageal reflux, were observed.

**Table 1:** Demographic information of the enrolled patients

Characters	Number (percentage, %)
Sex	
Male	183(59.8)
Female	123(40.2)
Facture location	
Upper extremity	96(31.4)
Lower extremity	203(66.3)
Pelvic/acetabular	7(2.3)
Comorbidity	
Yes	72(23.5)
No	233(76.1)
Anesthesia type	
General anesthesia	42(13.7)
Spinal/regional blocking	264(86.3)
Surgical type	
Open reduction with internal fixation	257(84.0)
Closed reduction with internal fixation	41(13.4)
Others	8(2.6)
Complications during hospitalization	
Yes	4(1.3)
No	302(98.7)

### Fasting time

The median preoperative fasting time for liquids and solids was 8 (5.2–12.9) and 19 (15.7–22) hours, respectively. The median postoperative fasting time for liquids and solids was 1 (0.5–1.9) and 2.8 (2.2–3.5) hours, respectively. The total perioperative fasting time for liquids and solids was 11 (8–15.7) and 23.7 (20.8–26.5) hours, respectively.

When the patients were divided according to preoperative carbohydrate loading compliance, the preoperative and total fasting times for liquids were significantly shorter in the compliance group (*P* < 0.05), whereas the postoperative fasting time for liquids showed no statistical significance (*P* > 0.05) (Table 2). The fasting time for solids was not significantly different (*P* > 0.05). The patients’ age, sex, fracture type, BMI, comorbidities, anesthesia type, surgery type, and surgical time also showed no significant differences between the two groups (*P* > 0.05).

**Table 2:** Comparison of perioperative fasting time between preoperative carbohydrate loading compliance and non-compliance groups

Fasting time	Compliance group (*N* = 219)	Non-compliance group (*N* = 77)	Z	*P*
Preoperative for liquids (hours, median, interquartile range)	6.8(4.3, 8.5)	15(12, 17.8)	11.243	< 0.0001
Preoperative for solids (hours, median, interquartile range)	18.8(15.6, 21.8)	18.8(15.8, 21.8)	0.227	0.821
Postoperative for liquids (hours, median, interquartile range)	1(0.6, 2)	0.7 (0.5, 1.5)	1.822	0.069
Postoperative for solids (hours, median, interquartile range)	2.9(2.3, 3.5)	2.7(2, 3.5)	1.428	0.153
Total for liquids (hours, median, interquartile range)	9.5(7.1, 12)	17.5(14.4, 20.5)	10.473	< 0.0001
Total for solids (hours, median, interquartile range)	23.7(20.9, 26.7)	23.3(20.5, 26)	0.727	0.467

### Subjective comfort analysis

In total, 215 (70.3%) and 227 (74.2%) patients had no thirst and hunger, respectively, during the perioperative period; 87 (28.4%) and 72 (23.5%) patients felt some thirst and hunger, respectively; and 4 (1.3%) and 7 (2.3%) patients had severe thirst and hunger, respectively. 22 (7.2%) and 3 (1.0%) patients suffered some and severe nausea, respectively. Whereas 14 (4.6) and 2 (0.7%) suffered some and severe vomiting. Logistic regression analysis was performed to identify the risk factors perioperative thirst and hunger. And length of pre- and post-operative fasting time for liquids and solids, total fasting time for liquids and solids, surgical type, anesthesia type, fracture type, sex, comorbidities and compliance rate of preoperative carbohydrate loading and abbreviated postoperative fasting were enrolled as potential risk factors. The results showed that the perioperative fasting time for liquids was an independent risk factor for perioperative hunger (*P* < 0.05). No risk factor was identified for perioperative thirst according to logistic regression analysis.

## Discussion

Many guidelines have already recommended abbreviated perioperative fasting for patients undergoing elective surgery, considering the benefits of improving patients’ subjective feelings, alleviating insulin resistance and reducing stress response [5–8, 13]. However, changes in real clinical practice are usually slow. With the development of the ERAS protocol, shortening of the perioperative fasting time and preoperative carbohydrate loading were conducted at our department. Analysis of 306 patients with fractures revealed a 71.6% compliance rate for preoperative carbohydrate loading, and 93.5% of patients resumed oral liquids within 2 h after surgery. Compared with non-complying patients who underwent preoperative carbohydrate loading, the preoperative and total fasting times for liquids were significantly shorter in the complying patients. More than 70% of patients had no thirst or hunger with the current management protocol.

There are many reasons for non-compliance with abbreviated preoperative fasting. Roughead et al. [21] classified patients’ compliance as active or passive based on whether the patients were required to make a direct contribution to achieve the compliance. Both education of doctors and nurses and cooperation by patients are needed for successful perioperative fasting management. Additionally, a long and difficult transition in real clinical practice can be predicted. A compliance rate of > 70% is usually considered satisfactory [22]. In this study, 71.6% of patients underwent preoperative carbohydrate loading. The result was acceptable, although it could still be improved. Nevertheless, the median preoperative fasting time for liquids was still long because of uncertainty of the starting time of the subsequent operation. Because solid food was still forbidden preoperatively on the day of surgery, the preoperative fasting time for solids was not improved.

The benefits of preoperative carbohydrate loading have been verified by many studies and include alleviating insulin resistance [2, 11, 23], improving patients’ subjective feelings [2, 4, 23, 24], and decreasing stress reaction [23–25]. Theoretically, a carbohydrate-enriched beverage provides not only provide liquid but also energy. In our study, the patients’ thirst and hunger were evaluated and the preoperative fasting time for liquids was an independent risk factor for perioperative hunger. This finding indicates that further shortening of the preoperative fasting time using a carbohydrate-enriched beverage was still needed because > 25% of patients still experienced different levels of thirst and/or hunger under the current management protocol.

There is no exact time to resume oral nutrition after elective surgery because of variations among the types of surgery performed and the patients’ tolerance [8]. Long periods of postoperative fasting are still being reported in the literature, especially for patients undergoing digestive tract operations [26, 27]. However, this is unnecessary according to a study by de Amorim et al. [28], who found that the median postoperative fasting time was reduced to 5.1 h. For surgeries not involving the gastrointestinal tract, the postoperative fasting time could be even shorter. Ford et al. [29] studied patients’ fasting after cardiothoracic surgery and showed that early oral hydration did not increase the incidence of nausea, vomiting, dysphagia, and aspiration pneumonia compared with usual care (a 6-h fast). Additionally, the number of patients with a high thirst level was significantly reduced in the early oral hydration group [29]. In our study, according to the recommendation of the Chinese guidelines, the median postoperative fasting time for liquids and solids was shortened to 1 and 2.8 h, respectively. No complications such as aspiration pneumonia were observed in this cohort, verifying the safety of this protocol.

This study had some limitations. Firstly, this was an observational study with no control group; thus we could not evaluate the improvement of patients’ subjective comfort under the abbreviated fasting protocol. Secondly, no biochemical test including stress biomarkers and insulin resistance were included due to the real clinical feature of the study. Thirdly, the preoperative fasting time for both liquids and solids was still long, so further improvement was needed. At last, the reasons for non-compliance for abbreviated fasting management were not analyzed, which would provide valuable information to further improve guideline adherence and be worth for further study.

## Conclusions

This study involved the implementation of an abbreviated perioperative fasting protocol based on current guidelines and analyzed patients’ compliance. Among 306 patients with fresh fractures, a 71.6% compliance rate for preoperative carbohydrate loading was achieved. The postoperative fasting time was shortened to 1 h for liquids and 2.8 h for solids. More than 70% of patients had no thirst or hunger perioperatively under the current protocol. However, the preoperative fasting time was still relatively long, and further abbreviating the preoperative fasting time would probably further improve patients’ subjective comfort according to our logistic regression analysis.

## Data Availability

The data and materials could be acquired by contacting the corresponding author with e-mail.

## Abbreviations

ERAS: Enhanced recovery after surgery
BMI: Body mass index
